# Evaluation of the Pharmacokinetics of the Pancreastatin Inhibitor PSTi8 Peptide in Rats: Integration of In Vitro and In Vivo Findings

**DOI:** 10.3390/molecules27020339

**Published:** 2022-01-06

**Authors:** Guru R. Valicherla, Roshan A. Katekar, Shailesh Dadge, Mohammed Riyazuddin, Anees A. Syed, Sandeep K. Singh, Athar Husain, Muhammad Wahajuddin, Jiaur R. Gayen

**Affiliations:** 1 Pharmaceutics & Pharmacokinetics Division, CSIR-Central Drug Research Institute (CSIR-CDRI), Lucknow 226031, India; gururaghava810@gmail.com (G.R.V.); roshankatekar123@gmail.com (R.A.K.); shailesh.dadge@gmail.com (S.D.); riyazuddinpharmacist@gmail.com (M.R.); syedaneesahmed5@gmail.com (A.A.S.); singhskbbd@gmail.com (S.K.S.); atharhusain001@gmail.com (A.H.); wahajuddin@gmail.com (M.W.); 2 Academy of Scientific and Innovative Research, Ghaziabad 201002, India; 3 Pharmacology Division, CSIR-Central Drug Research Institute (CSIR-CDRI), Lucknow 226031, India

**Keywords:** antidiabetic drugs, PSTi8, in vitro ADME, in vivo pharmacokinetics, bioavailability

## Abstract

PSTi8 is a pancreastatin inhibitory peptide that is effective in the treatment of diabetic models. This study investigates the pharmacokinetic (PK) properties of PSTi8 in Sprague Dawley rats, for the first time. In vitro and in vivo PK studies were performed to evaluate the solubility, stability in plasma and liver microsomes, plasma protein binding, blood–plasma partitioning, bioavailability, dose proportionality, and gender difference in PK. Samples were analyzed using the validated LC-MS/MS method. The solubility of PSTi8 was found to be 9.30 and 25.75 mg/mL in simulated gastric and intestinal fluids, respectively. The protein binding of PSTi8 was estimated as >69% in rat plasma. PSTi8 showed high stability in rat plasma and liver microsomes and the blood–plasma partitioning was >2. The bioavailability of PSTi8 after intraperitoneal and subcutaneous administration was found to be 95.00 ± 12.15 and 78.47 ± 17.72%, respectively, in rats. PSTi8 showed non-linear PK in dose proportionality studies, and has no gender difference in the PK behavior in rats. The high bioavailability of PSTi8 can be due to high water solubility and plasma protein binding, low clearance and volume of distribution. Our in vitro and in vivo findings support the development of PSTi8 as an antidiabetic agent.

## 1. Introduction

In the 21st century, diabetes is one of the rapidly growing health challenges in the world. Globally, there are 537 million people living with diabetes in 2021, of which around 90% of the cases accounted for type 2 diabetes [1,2]. To date, existing drugs cannot completely cure type 2 diabetes, but they can manage glucose levels and prevent complications associated with the different organs [3,4]. There is a strong interest in the discovery and development of more effective drugs for the treatment of type 2 diabetes.

PSTi8 (PEGKGEQEHSQQKEEEEEMAV-amide), pancreastatin (PST) inhibitory peptide, was discovered and developed by the CSIR-Central Drug Research Institute (CSIR-CDRI, Indian patent published 201611010438) [5]. PSTi8 has 21 amino acids with amidation at the C-terminal. The isoelectric point and charge at pH 7 of the PSTi8 peptide, were observed as ≈4 and −5, respectively [6]. The PSTi8 peptide showed potent antidiabetic activity in several preclinical diabetes and insulin resistance (IR) models, such as diet-induced diabetes and IR mice, db/db mice, diet-induced IR postmenopausal rats, dexamethasone induced type 2 diabetes mice and chronic hyperinsulinemia mice [5,7,8,9,10]. It is reported that PSTi8 showed antidiabetic activity by stimulating insulin signaling in the liver via AKT and PKC pathways, and inhibiting stress signaling in adipose via MAPK and NOX3-JNK pathways [5,7,9]. PSTi8 showed a decrease in the gluconeogenesis process in the liver, by downregulating gene expression levels, such as phosphoenolpyruvate carboxykinase and glucose-6-phosphatase [5,7,8]. The PSTi8 peptide was found to be safe and non-toxic with acute and chronic dose toxicity studies in C57Bl/6 mice [8]. PSTi8 is a highly potent antidiabetic peptide and the chronic dose used for antidiabetic activity in mice and rat models was 2 and 1 mg/Kg, respectively [5,7].

Prior to the commencement of clinical studies, a comprehensive assessment of preclinical in vitro and in vivo PK information is a regulatory prerequisite for a better understanding of the efficacy, safety and toxicity profiles of investigational new drugs. In the current study, we investigate the in vitro and in vivo PK studies of PSTi8 for the first time in rats. In vitro studies, such as solubility, blood partitioning, plasma protein binding and stability in plasma and liver microsomes, were conducted to understand the disposition of PSTi8. PSTi8 was administered in different routes to evaluate bioavailability and PK behavior in male Sprague Dawley (SD) rats. The dose proportionality study was performed to determine the linear/non-linear PK pattern in male SD rats. Gender difference in PK studies was performed to perceive the differences in the PK parameters in male and female SD rats. The in vitro and in vivo PK knowledge obtained from this work is essential for the development of the PSTi8 peptide as a safe and effective antidiabetic drug.

## 2. Results and Discussion

### 2.1. LC-MS/MS Bioanalytical Method

In the recent report, we have described the LC-MS/MS method development and validation of PSTi8 in mice plasma [6]. No interference from the rat plasma matrix at the retention times of PSTi8 or diprotin A (internal standard, IS) was perceived, as shown in Figure S1. The linearity was accomplished using the area ratios of PSTi8 and IS across the calibration range of 5–1000 ng/mL concentrations with r^2^ ≥ 0.997 (*n* = 6). The mean linear regression equation for PSTi8 in rat plasma was y = 0.00034x + 0.0059 with a 1/X^2^ weighing factor. The intra- and inter-day precision and accuracy results of the quality controls (QCs) were found within the acceptable criteria (Table S1). The intra- and inter-day accuracy of PSTi8 were found between 100.50–105.30% and 99.88–103.98%, respectively. The intra- and inter-day precision of PSTi8 were found between 2.29–3.64% and 5.73–8.43%, respectively. The accuracy and precision results of the stability studies of PSTi8 were found within the criteria of an acceptable range. The PSTi8 peptide was found to be stable in rat plasma with diverse storage conditions (Table S2). We have successfully utilized the LC-MS/MS bioanalytical assay of the PSTi8 peptide for the evaluation of in vitro and in vivo PK properties. The plasma samples of PSTi8 collected after PK studies in rats were quantified by the validated assay together with the QC samples.

### 2.2. Solubility Study

The equilibrium solubility of PSTi8 in simulated gastric fluid (SGF) and simulated intestinal fluid (SIF) was found to be 9.30 ± 0.40 and 25.75 ± 0.88 mg/mL, respectively.

### 2.3. Plasma Stability

**Figure 1:**
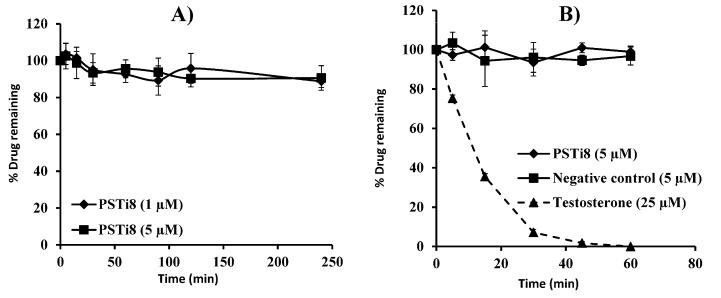
Stability of PSTi8 peptide in (**A**) rat plasma and (**B**) rat liver microsomes. Data are represented in *n* = 3 with a mean ± SD.

Plasma stability plays an important role in drug discovery and development. Unstable compounds tend to have rapid clearance and a short half-life, resulting in poor in vivo performance. The plasma stability profiles of PSTi8 are shown in Figure 1A. The stability of PSTi8 at 1 and 5 µM concentrations was found to be 88.80 ± 3.39 and 90.56 ± 6.65%, respectively, at 4 h in rat plasma.

### 2.4. Microsomal Metabolic Stability

Microsomal metabolic stability of PSTi8 was performed at a 5 µM concentration in rat liver microsomes (RLM). Different RLM and nicotinamide adenine dinucleotide phosphate (NADPH) concentrations were tried to optimize the method. The PSTi8 peptide was found to be stable in RLM (Figure 1B) and has no degradation in the negative control experiment (without NADPH). The half-life of positive control (25 µM testosterone) was observed within the acceptable in-house limits.

### 2.5. Plasma Protein Binding

It is well known that only an unbound drug can reach the target site and exert its pharmacological action. Therefore, it is worthy to evaluate the unbound drug for identifying the parameters that affect the drug PK and pharmacodynamics. Ultrafiltration and equilibrium dialysis are common approaches for plasma protein binding estimation, but they have some drawbacks, such as nonspecific adsorption of the drugs to the device parts [11]. Therefore, a modified charcoal adsorption method was utilized for the protein binding estimation of PSTi8 in rat plasma. The modified charcoal adsorption method depends on the kinetics of charcoal adsorption and works in non-equilibrium conditions [12]. The plasma protein binding of PSTi8 at 2 µM and 5 µM was estimated as 73.63 ± 0.91 and 69.34 ± 8.47 %, respectively, which suggest that it has high plasma protein binding. The plasma protein binding has a significant influence on drug PK parameters, such as clearance (CL/F) and apparent volume of distribution (V_d_/F) [13]. The high plasma protein binding of PSTi8 explains the low V_d_/F and CL/F values observed in in vivo PK data.

### 2.6. Blood–Plasma Partitioning

The red blood cells (RBC): plasma partition coefficient (K_RBC/PL_) values of PSTi8 at 0.206 and 0.412 µM concentrations were found to be 2.46 ± 0.44 and 2.32 ± 0.76, respectively. The rate of RBC partitioning of PSTi8 was found to be rapid, as there was no significant difference (*p* > 0.05, Table 1) between K_RBC/PL_ at various time points at the two concentrations.

### 2.7. Evaluation of Preclinical PK Studies

#### 2.7.1. Bioavailability Assessment in Different Routes of Administration

**Figure 2:**
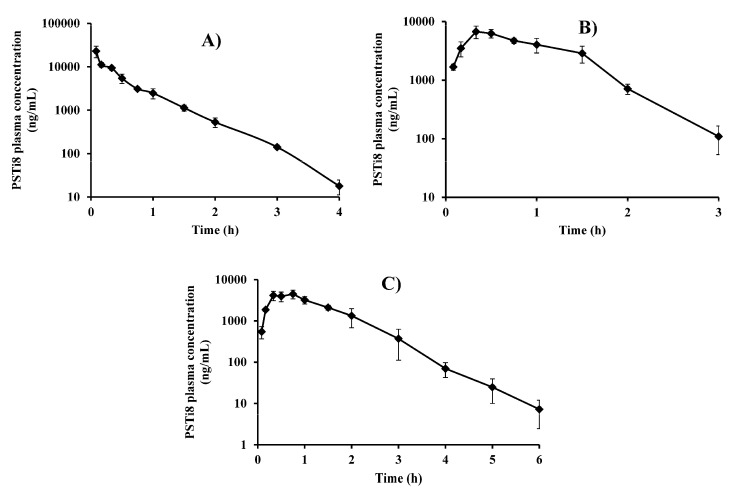
Pharmacokinetic studies of the PSTi8 peptide in different routes of administration in male SD rats. Plasma concentration against time profiles of the PSTi8 peptide at a dose of 5 mg/kg, after (**A**) i.v., (**B**) i.p. and (**C**) s.c. administration to each group. Data are represented in *n* = 6 with the mean ± SD.

The acute (5 mg/kg, intraperitoneal) administration of the PSTi8 peptide showed potential antidiabetic activity in postmenopausal IR rats [7]. Therefore, we used the same dose for the PK determination in different routes of administration. The PK profiles and parameters of PSTi8 peptide after intravenous (i.v.), intraperitoneal (i.p.) and subcutaneous (s.c.) dosing in male SD rats are represented in Figure 2A–C and Table 2. The maximum plasma concentration (C_max_) was determined as 6047.77 ± 348.49, 4105.49 ± 888.30 and 30193.35±14562.01 µg/L concentrations after i.p., s.c. and i.v. administration of the PSTi8 peptide, respectively. The time to reach C_max_ (T_max_) of PSTi8 was found to be 0.48 and 0.60 h in i.p. and s.c. PK studies, respectively. The elimination half-life (t_1/2_) of PSTi8 was observed as 0.44 ± 0.10, 0.42 ± 0.004 and 0.25 ± 0.18 h, following i.p., s.c. and i.v. dosing, respectively. The PSTi8 peptide rapidly reached its C_max_ and was eliminated with low t_1/2_. The V_d_/F of PSTi8 after i.v., i.p. and s.c. administration was observed as 0.20 ± 0.11, 0.39±0.06 and 0.46 ± 0.09 L/kg, respectively. V_d_ was less than 0.668 L/kg (total body water) in rats, which specifies that the PSTi8 peptide has a low tissue distribution [14]. The CL/F of PSTi8 was observed as 0.60 ± 0.10, 0.62 ± 0.08 and 0.77 ± 0.16 L/h/kg after i.v., i.p. and s.c. dosing, respectively. The CL values of PSTi8 were lower than 1.74 L/h/kg (hepatic plasma flow), which indicates that PSTi8 peptide has low extraction. After i.p., s.c., and i.v. administration, the area under curve (AUC) of PSTi8 was determined as 8099.23 ± 1035.68, 6690.13 ± 1510.57 and 8525.66 ± 1604.03 h*µg/L, respectively. The absolute bioavailability of the PSTi8 peptide for i.p. and s.c. was 95 ± 12.15 and 78.47 ± 17.72%, respectively, which suggests that it has a high bioavailability. The high aqueous solubility, low V_d_/F, low CL/F and high plasma protein binding properties of PSTi8, can be responsible for the high bioavailability after i.p. and s.c. dosing in rats. The PSTi8 bioavailability for i.p. was reported as 52% in mice [6], which demonstrates that the PSTi8 peptide has high and moderate i.p. bioavailability in rats and mice, respectively. The difference in the bioavailability of PSTi8 in rats and mice can be due to the physiological differences in these species. After i.p. treatment, PSTi8 showed higher plasma concentrations, C_max_, AUC and bioavailability than the s.c. treatment. Therefore, we performed the dose proportionality and gender difference studies for the PSTi8 peptide using the i.p. treatment in rats.

#### 2.7.2. Dose Proportionality PK Studies

**Figure 3:**
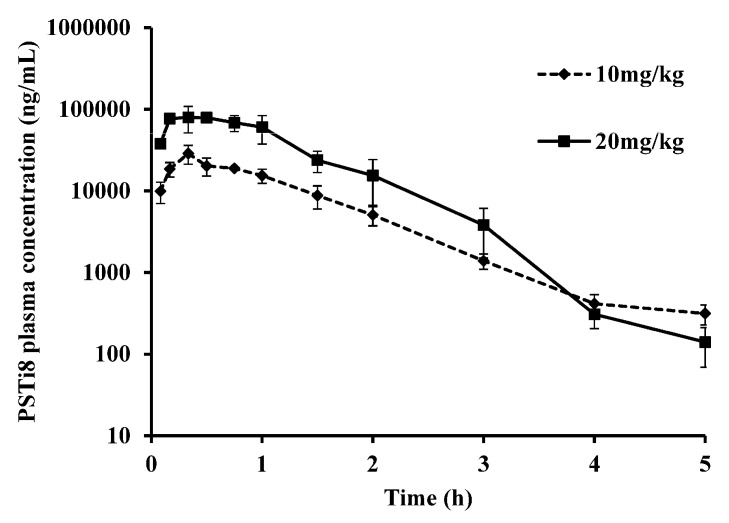
Dose proportionality pharmacokinetic studies of the PSTi8 peptide in male SD rats. Plasma concentration against time profiles of PSTi8 peptide after i.p. administration at 10 and 20 mg/kg to each group. Data are represented in *n* = 6 with the mean ± SD.

The PSTi8 peptide is safe and non-toxic at acute (50 and 250 mg/kg, i.p.) high doses in mice [8]. In order to understand the dose proportionality PK behavior, PSTi8 was administered i.p. in male SD rats at a dose of 10 and 20 mg/kg to each group. The plasma concentration vs. time profiles and PK parameters of PSTi8 after i.p. treatment in male SD rats at 10 and 20 mg/kg, are represented in Figure 3 and Table 3. For the nominal i.p. doses, 1:2 proportion, the C_max_ and AUC values were found in the ratio of 1.00:3.52 and 1.00:3.44, respectively (Table 3). PSTi8 undergoes non-linear pharmacokinetics in the dose proportionality study. To understand the non-linear pharmacokinetics of PSTi8, a comprehensive study of a larger number of doses and peptide clearance mechanisms are warranted.

#### 2.7.3. Gender Differences in PK

**Figure 4:**
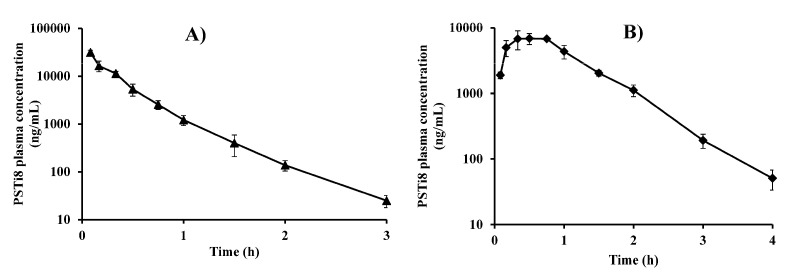
Pharmacokinetic studies of the PSTi8 peptide in female SD rats. Plasma concentration against time profiles of the PSTi8 peptide after at a dose of 5 mg/kg (**A**) i.v. and (**B**) i.p. administration to each group. Data are represented in *n* = 6 with the mean ± SD.

The gender disparity in the efficacy and toxicity profiles of drugs, depends primarily on the PK and metabolism pattern. Due to the differences in the physiology of men and women, there is a difference in the pharmacokinetics of drugs [15,16]. The PSTi8 was administered at a dose of 5 mg/kg i.v. and i.p. in female SD rats to each group. The PK profiles and parameters of PSTi8 after i.v. and i.p. treatment in female SD rats are represented in Figure 4A,B and Table 4. After i.p. dosing of PSTi8 in female SD rats, the C_max_ (7004.16 ± 216.23 µg/L) was rapidly reached at 0.48 h (t_max_) and then eliminated with t_1/2_ of 0.32 ± 0.02 h. The V_d_/F for i.v. and i.p. were found to be 0.12 ± 0.01 and 0.26 ± 0.01 L/kg, respectively. The CL/F values of PSTi8 after i.v. and i.p. administration were observed as 0.53 ± 0.10 and 0.57 ± 0.04 L/h/kg, respectively. After i.p. treatment, the AUC of PSTi8 was found to be 9983.31 ± 523.03 h*µg/L in female rats. After i.v. administration, C_max_ and AUC of PSTi8 were observed as 42585.13 ± 3706.02 µg/L and 9590.04 ± 1747.10 h*µg/L, respectively, in female rats. The bioavailability of PSTi8 in female SD rats for i.p. was 91.92 ± 6.14%. There was no significant difference observed in the PK parameters and bioavailability of the PSTi8 peptide after i.p. and i.v., in male (Table 2) and female SD rats (Table 4).

## 3. Materials and Methods

### 3.1. Chemicals

PSTi8 (2427 Da and >98% purity) was synthesized from the Life Tein LLC (Somerset, NJ, USA). LC-MS/MS grade methanol, dextran-coated charcoal, Diprotin A (IS), Dulbecco’s phosphate buffer saline (DPBS), potassium ethylene diamine tetraacetic acid (K_2_EDTA), NADPH, formic acid and magnesium chloride were purchased from Sigma Aldrich (Bengaluru, India). Diethyl ether was purchased from TKM pharma (Hyderabad, India). Medium Anion Exchange (MAX) Oasis cartridges were procured from Waters Corporation (Milford, MA, USA).

### 3.2. Animals

Young, healthy SD male and female rats (200 ± 20 g, 9 ± 1 weeks) were obtained from the Laboratory Animal Facility of CSIR-CDRI. Animals were accommodated in ventilated cages at 50 ± 10% RH and RT 24 ± 2 °C, with a regular 12 h light–dark cycle. Animal studies were accomplished with prior approval from the Institutional Animal Ethics Committee of CSIR-CDRI (IAEC approval no. IAEC/2012/91). Blood was withdrawn from the retro-orbital plexus of rats under light ether anesthesia, using an anticoagulant (K_2_EDTA) in the microcentrifuge tubes. The tubes were centrifuged at 3000 g for 10 min, and the plasma was separated and kept at −80 °C in a freezer until analysis.

### 3.3. LC-MS/MS Analysis

A QTRAP-4000 AB Sciex (SCIEX, Concord, ON, Canada) mass spectrometer using an electrospray ionization source tandem with the Shimadzu UFLC system was utilized for the quantitative analysis. Quantification was performed for PSTi8 and IS with positive ionization mode, using prominent selected reaction monitoring transitions as 607.80/771.20 and 342.20/229.10 *m*/*z*, respectively. The chromatographic separation of PSTi8 and IS was accomplished on a Phenomenex Aqua 5µ 125A (250 × 4.6 mm) column using an isocratic mode of mobile phase (methanol:0.1 % formic acid, 50:50 % *v*/*v*) with 0.4 mL/min flow rate. The analysis run time was 10 min and the injection volume was 10 μL for each sample. Analyst 1.6 software was used to regulate the LC-MS/MS instrument and for data quantification. The plasma sample extraction involved the SPE procedure using MAX cartridges, as previously described [6]. The LC-MS/MS bioanalytical assay of PSTi8 peptide was validated in rat plasma as per the USFDA bioanalytical method validation guidance [17]. The bioanalytical method development and validation of PSTi8 in mice plasma was recently reported [6]. The bioanalytical validation and stability studies were conducted in rat plasma, as described earlier [6].

### 3.4. Solubility Study

The solubility of PSTi8 peptide was conducted using the equilibrium solubility method in SGF (pH 1.2) and SIF (pH 7.4) [18]. A total of 50 mg of PSTi8 was added to silanized glass vials containing 1 mL of buffer, and incubated for 1 h at 37 °C in a shaking water bath to attain equilibrium. After incubation, the samples were centrifuged for 10 min at 12,000 g, and supernatant was processed through SPE procedure and used for LC-MS/MS analysis.

### 3.5. Plasma Stability Study

Fresh rat plasma (1 mL) was used and pre-incubated in a shaking water bath at 37 °C for 10 min. PSTi8 was added to rat plasma at a final concentration of 1 or 5 µM and then incubated for 1 h at 37 °C in a shaking water bath. A total of 100 µL samples were aliquoted at predetermined time points (0, 5, 15, 30, 60, 90, 120 and 240 min), followed by SPE processing and analyzed using the validated LC-MS/MS method. The plasma stability was determined as the % drug remaining at different time points relative to the drug at 0 min [19].

### 3.6. Microsomal Metabolic Stability Study

RLM was prepared from SD rats from our lab, as previously reported [18]. In vitro metabolic stability of PSTi8 was determined using RLM. The reaction milieu was prepared by adding 50 mM Tris buffer (pH 7.4), 40 mM MgCl_2_ pre-incubated at 37 °C along with RLM (0.5 mg/mL) and spiked at a concentration (5 µM) of PSTi8. The reaction was started with NADPH (1 mM) and without NADPH (negative control using Tris buffer). A total of 100 µL samples were collected at 0, 5, 15, 30, 45 and 60 min, and the reaction was quenched with liquid nitrogen. Samples were processed with the SPE procedure and analyzed using the LC-MS/MS method. Negative control was used to assess the peptide stability in the reaction milieu and the positive control (Testosterone, 25 µM) was incubated to obtain the microsomal activity.

### 3.7. Plasma Protein Binding Study

The protein binding study of the PSTi8 peptide was conducted in rat plasma using the modified charcoal adsorption method. The suspension of dextran-coated charcoal (0.66%) was prepared in the DPBS buffer (pH 7.4) and stirred overnight (18 h), before the study. PSTi8 (2 µM and 5 µM) was prepared in 2.5 mL rat plasma and pre-incubated at 37 ± 0.5 °C for 15 min in a shaking water bath. The exact volume of the charcoal suspension, the same as the plasma, was centrifuged at 3000 g for 15 min to separate DPBS. The supernatant was decanted and the pre-incubated plasma was added to the charcoal pellet-containing tubes. Plasma-containing charcoal tubes were incubated under continuous stirring up to 4 h. A total of 200 µL samples were collected at the time points of 0, 5, 10, 20, 30, 45, 60, 90, 120 and 180 min, and then centrifuged at 11,000 g for 2.5 min. A total of 100 µL supernatant of the centrifuged sample was separated, extracted using SPE and analyzed with LC-MS/MS. The % peptide remaining in the supernatant plasma sample versus time points data was evaluated using the i.v. bolus two-compartment analysis model on Phoenix 6.3 WinNonlin (Pharsight Corporation, Saint Louis, MO, USA) [20,21]. The model is defined by the bi-exponential Equation (1):

(1) B(t)=A1exp(−αt)+A2exp(−βt)

where *B*(*t*) % is the bound peptide remaining at time *t* and where *A*1, *A*2, *α* and *β* are constants.

### 3.8. Blood–Plasma Partitioning Study

Fresh blood was collected from SD rats and used for the blood–plasma partitioning study. The blood was pre-incubated in a shaking water bath for 10 min at 37 ± 0.5 °C. The blood–plasma partitioning study was performed for PSTi8 at 0.206 µM (0.5 µg/mL) and 0.412 µM (1 µg/mL) concentrations. During the study, the fresh rat plasma was separated from the same batch of whole blood. During the incubation time course, 250 µL aliquots of incubated whole blood samples were collected at different time points (0, 15, 30 and 60 min) and centrifuged at 10,000 g for 5 min at RT to obtain the plasma. At each time point, 100 µL aliquot of the reference plasma sample was collected. Both, 100 µL aliquot of generated plasma and reference plasma were processed and analyzed using the LC-MS/MS method. For the determination of hematocrit (*Hc*), heparinized whole blood (1.0 mL) was taken in a hematocrit tube and centrifuged at 2000 g for 30 min. The *Hc* for the whole blood was calculated by using Equation (2) [21,22].

(2) $$Hc=\frac{\mathrm{Volume}\;\mathrm{of}\;\mathrm{blood}\;\mathrm{cells}}{\mathrm{Total}\;\mathrm{blood}\;\mathrm{volume}}$$

The *K_RBC/PL_* for PSTi8 was determined from the following Equation (3):

(3) $$K_{RBC/PL}=\frac{1}{H}\left[\frac{I_{Ref}}{I_{Pl}}-1\right]+1$$

where *I_Ref_* is the peak area ratio of reference plasma, and *I_Pl_* is the peak area ratio of equilibrating plasma.

### 3.9. Preclinical PK Evaluation

#### 3.9.1. Bioavailability Assessment in Different Routes of Administration

The i.v., i.p. and s.c. PK studies were conducted for PSTi8 peptide in male SD rats. Rats were distributed into three groups and each group contained 6 animals. A total of 5 mg/kg bolus dose of PSTi8 solution was administered i.v., i.p. and s.c. to each group (*n* = 6). During i.v. dosing, PSTi8 solution was injected into rats via the lateral tail vein.

#### 3.9.2. Dose Proportionality in PK Studies

Male SD rats were utilized in this study and distributed into two groups (each group contained 6 animals). To each group, PSTi8 was administered i.p. at a dose of 10 and 20 mg/kg in rats.

#### 3.9.3. Gender Differences in PK

Female SD rats were utilized and distributed into two groups (each group contained 6 animals). To each group, a 5 mg/kg dose of PSTi8 was administered i.v. and i.p. for PK determination.

#### 3.9.4. Sampling Schedule

After dosing in rats, blood (250 µL) samples were collected in EDTA containing microcentrifuge tubes at 0.08, 0.16, 0.33, 0.50, 0.75, 1, 2, 3, 4, 5 and 6 h time points. Plasma was separated from the blood after centrifugation and kept in a −80 °C freezer for LC-MS/MS analysis.

#### 3.9.5. PK Data Analysis

The PK parameters of PSTi8 were estimated with a one-compartmental PK model in Phoenix WinNonlin. The i.p. and s.c. PK were best described by a one-compartment first-order PK model without lag time. The i.v. PK was well explained by a one-compartment model with a bolus input and first-order elimination rate. The PK parameters include the elimination constant (K_10_), absorption constant (K_01_), T_max_, elimination half-life (K_10_-HL), absorption half-life (K_01_-HL), C_max_, AUC (stands for model derived AUC_0-∞_), CL/F, mean residence time (MRT) and V_d_/F. The bioavailability of PSTi8 was determined using the ratio between the AUC from i.p. or s.c. and i.v. routes, after normalizing the dose.

### 3.10. Statistical Analysis

All the data are represented as mean ± SD, except T_max_, which is presented as the median. Statistical analysis was performed with the unpaired Student’s *t*-test using the GraphPad Prism software version 9.0 (GraphPad Software, La Jolla, CA, USA). A *p*-value of <0.05 was considered as statistically significant.

## 4. Conclusions

This is the first study investigating the in vitro and in vivo pharmacokinetics of the PSTi8 peptide in rats. The PSTi8 peptide was found to be stable in rat plasma and liver microsomes. The high bioavailability of PSTi8 for i.p. administration in rats can be due to high aqueous solubility, high plasma protein binding, low clearance and low volume of distribution. The PSTi8 peptide exhibited non-linear PK in dose proportionality studies, and has no gender differences in PK between male and female SD rats. Further studies are required to investigate the PSTi8 peptide disposition mechanism involved in the nonlinear pharmacokinetics in dose proportionality studies, species differences in PK and multiple dose PK behavior. The in vitro and in vivo PK findings support the development of the PSTi8 peptide as a safe and effective antidiabetic drug.

## Figures and Tables

**Table 1:** K_RBC/PL_ of PSTi8 in rat whole blood at 0.206 µM and 0.412 µM. Data are presented as the mean ± SD with *n* = 3.

Time (min)	K_RBC/PL_		*p*-Value
	Conc. (0.206 µM)	Conc. (0.412 µM)	
0	1.83 ± 0.48	2.23 ± 0.02	0.22
15	2.56 ± 0.25	2.94 ± 0.62	0.38
30	1.88 ± 0.25	2.55 ± 0.65	0.17
45	2.05 ± 0.12	2.19 ± 0.12	0.22
60	2.46 ± 0.45	2.33 ± 0.77	0.81

**Table 2:** Pharmacokinetic parameters of the PSTi8 peptide after i.v., i.p. and s.c. administration at 5 mg/kg in male SD rats. Data are presented as the mean ± SD with *n* = 6.

Parameters		PSTi8 5 mg/kg	
	i.v.	i.p.	s.c.
AUC (h*µg/L)	8525.66 ± 1604.03	8099.23 ± 1035.68	6690.13 ± 1510.57
C_max_ (µg/L)	30193.35 ± 14562.01	6047.77 ± 348.49	4105.49 ± 888.30
T_max_ (h)	-	0.48	0.60
CL/F (L/h/kg)	0.60 ± 0.10	0.62 ± 0.08	0.77 ± 0.16
V_d_/F (L/kg)	0.20 ± 0.11	0.39 ± 0.06	0.46 ± 0.09
K_01_-HL (h)	-	0.26 ± 0.09	0.41 ± 0.01
K_10_-HL (h)	0.25 ± 0.18	0.44 ± 0.10	0.42 ± 0.004
K_01_ (1/h)	-	2.90 ± 0.89	1.68 ± 0.02
K_10_ (1/h)	3.79 ± 2.19	1.63 ± 0.33	1.66 ± 0.01
MRT (h)	0.36 ± 0.26	-	-
Bioavailability (%)	-	95.00 ± 12.15	78.47 ± 17.72

**Table 3:** Pharmacokinetic parameters of the PSTi8 peptide after i.p. administration at two doses of 10 and 20 mg/kg in male SD rats. Data are represented as the mean ± SD with *n* = 6.

Parameter	i.p. PSTi8 10 mg/kg	i.p. PSTi8 20 mg/kg
AUC (h*µg/L)	32,384.05 ± 3270.55	111,486.37 ± 30,126.00
C_max_ (µg/L)	24,272.74 ± 2681.30	85,606.83 ± 15,031.65
T_max_ (h)	0.38	0.38
CL/F (L/h/kg)	0.31 ± 0.03	0.19 ± 0.06
V_d_/F (L/kg)	0.26 ± 0.09	0.15 ± 0.03
K_01_-HL (h)	0.15 ± 0.05	0.14 ± 0.03
K_10_-HL (h)	0.59 ± 0.23	0.56 ± 0.09
K_01_ (1/h)	4.89 ± 1.74	4.94 ± 0.97
K_10_ (1/h)	1.33 ± 0.60	1.26 ± 0.18

**Table 4:** Pharmacokinetic parameters of the PSTi8 peptide after i.v. and i.p. administration at 5 mg/kg in female SD rats. Data are presented as the mean ± SD with *n* = 6.

Parameter	i.v. PSTi8 5 mg/kg	i.p. PSTi8 5 mg/kg
AUC (h*µg/L)	9590.04 ± 1747.10	8815.40 ± 588.58
C_max_ (µg/L)	42,585.13 ± 3706.02	7004.16 ± 216.23
T_max_ (h)	-	0.48
CL/F (L/h/kg)	0.53 ± 0.10	0.57 ± 0.04
V_d_/F (L/kg)	0.12 ± 0.01	0.26 ± 0.01
K_01_-HL (h)	-	0.32 ± 0.02
K_10_-HL (h)	0.16 ± 0.02	0.32 ± 0.02
K_01_ (1/h)	-	2.14 ± 0.12
K_10_ (1/h)	4.50 ± 0.48	2.19 ± 0.18
MRT (h)	0.22 ± 0.02	-
Bioavailability (%)	-	91.92 ± 6.14

## Data Availability

Data supporting the reported results will be available from the corresponding author (Jiaur R. Gayen).

## Supplementary Materials

The following supporting information can be downloaded at, Figure S1: Representative SRM chromatograms in (A) blank rat plasma, (B) zero sample (rat plasma spiked with IS), (C) rat plasma spiked with PSTi8 (LLOQ) and IS, and (D) 5 min sample of i.v. pharmacokinetic study in male SD rats with IS; Table S1: Precision and accuracy data of QC samples PSTi8 in rat plasma; Table S2: PSTi8 peptide stability in rat plasma in different storage conditions at 3 QC levels.

## Sample Availability

Samples of the compound are not available from the authors.
