# Supplementary material for: Evaluation of the Pharmacokinetics of the Pancreastatin Inhibitor PSTi8 Peptide in Rats: Integration of In Vitro and In Vivo Findings
Source: Molecules. 2022 Jan 6;27(2):339. doi: 10.3390/molecules27020339 (PMC8780964; doi:10.3390/molecules27020339)
Supplement: Supplementary file 1 [file molecules-27-00339-s001.zip › molecules-1477178-supplementary.pdf]

Supplementary File

Evaluation of the pharmacokinetics of pancreastatin inhibitor PSTi8 peptide in rats: integration of *in vitro* and *in vivo* findings

Guru R. Valicherla[1,3], Roshan A. Katekar[1,3], Shailesh Dadge[1], Mohammed Riyazuddin[1], Anees A. Syed[1,3], Sandeep K. Singh[1,3], Athar Husain[1,3], Muhammad Wahajuddin[1,3], Jiaur R. Gayen[1,2,3*]

[1]Pharmaceutics & Pharmacokinetics Division, [2]Pharmacology Division, CSIR-Central Drug Research Institute (CSIR-CDRI), Lucknow, 226031, India

[3]Academy of Scientific and Innovative Research, Ghaziabad 201002, India

Running title: *In vitro* and *in vivo* PK of PSTi8 in rats

* Correspondance at:

Dr. Jiaur R. Gayen,

Principal Scientist

Pharmaceutics & Pharmacokinetics Division,

CSIR-Central Drug Research Institute

Sitapur road, Lucknow-226031, India

E-mail: jr.gayen@cdri.res.in

Supplementary Tables:

Table S1. Precision and accuracy data of QC samples PSTi8 in rat plasma. The data are represented as mean±SD.

**Level**	**Nominal concentration (ng/mL)**	**Intra-day(n=6)**			**Inter-day (n=18)**		
		**Observed concentration (ng/mL)**	**Precision (%)**	**Accuracy (%)**	**Observed concentration (ng/mL)**	**Precision (%)**	**Accuracy (%)**
LLOQ	5	5.26 ± 0.12	2.37	105.30	5.20 ± 0.30	5.82	103.98
LQC	30	30.40 ± 0.93	3.05	101.25	30.56 ± 1.75	5.73	101.86
MQC	400	402.09 ± 14.65	3.64	100.50	407.67 ± 24.13	5.92	101.90
HQC	800	809.41 ± 18.56	2.29	101.15	799.14 ± 67.38	8.43	99.88

Table S2. PSTi8 peptide stability in rat plasma in different storage conditions at 3 QC levels. The data are represented as mean±SD with n=6.

Stability	Nominal concentration (ng/mL)	Observed mean concentration (ng/mL)	Precision (%)	Accuracy (%)
0 h (for all)	30	30.68 ± 2.34	7.61	102.34
	400	406.22 ± 29.17	7.18	101.56
	800	813.59 ± 83.51	10.26	101.70
Autosampler (4 °C, 12 h)	30	33.77 ± 0.50	1.49	112.60
	400	376.55 ± 36.86	9.79	94.13
	800	799.15 ± 26.68	3.34	99.90
Bench-top (2 h)	30	28.88 ± 1.05	3.65	96.27
	400	384.77 ± 37.59	9.77	96.17
	800	853.38 ± 73.43	8.60	106.70
Bench-top (6 h)	30	31.04 ± 0.53	1.71	103.47
	400	371.98 ± 19.28	5.18	93.00
	800	783.71 ± 82.95	10.58	97.97
Three freeze-thaw cycles	30	30.39 ± 3.64	11.97	101.30
	400	425.11 ± 5.03	1.18	106.30
	800	870.13 ± 20.75	2.38	108.77
Long term (-80°C, 30 days)	30	33.01 ± 2.55	7.72	110.13
	400	411.52 ± 24.99	6.07	102.87
	800	864.61 ± 48.26	5.58	108.03

Supplementary Figures:

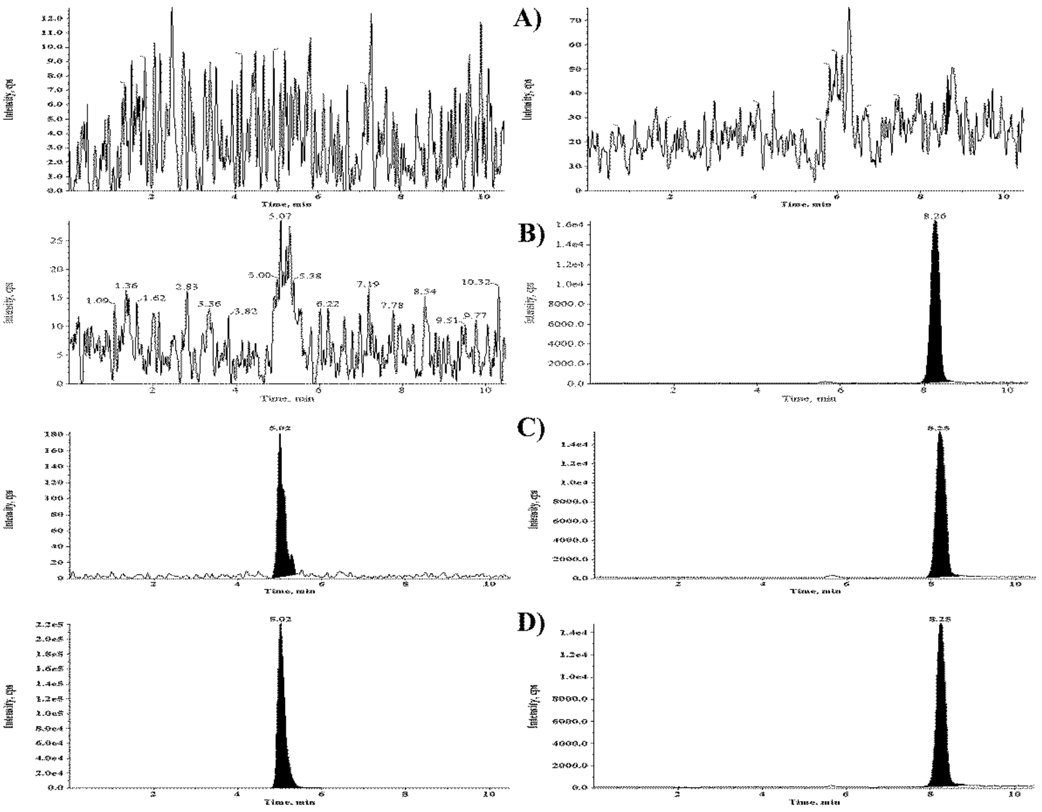

Figure S1. Representative SRM chromatograms in (**A**) blank rat plasma, (**B**) zero sample (rat plasma spiked with IS), (**C**) rat plasma spiked with PSTi8 (LLOQ) and IS, and (**D**) 5 min sample of *i.v.* pharmacokinetic study in male SD rats with IS.
